# Adaption and validation of the childbirth experience questionnaire (CEQ-SK) in Slovakia

**DOI:** 10.18332/ejm/160973

**Published:** 2023-03-14

**Authors:** Lena Henriksen, Janka Debrecéniová, Anna Hrabovská, Šarlota Pufflerová, Ellen Blix

**Affiliations:** 1 Department of Nursing and Health Promotion, Oslo Metropolitan University, Oslo, Norway; 2 Division of General Gynaecology and Obstetrics, Oslo University Hospital, Oslo, Norway; 3 Citizen, Democracy and Accountability, Bratislava, Slovakia

**Keywords:** Childbirth experience questionnaire, childbirth experiences, CEQ, validation, Slovakia

## Abstract

**INTRODUCTION:**

Using validated and reliable instruments to examine women’s birth experiences is important to ensure respectful care. There is a lack of validated instruments for evaluating childbirth care in the Slovak context. In this study, we aimed to adapt and validate the childbirth experience questionnaire (CEQ) in Slovakia (CEQ-SK).

**METHOD:**

The CEQ-SK was developed and modified from the English version of the CEQ/CEQ2. Face validity was tested in two pre-tests. A convenience sample, recruited through social media, included 286 women who had given birth within the last six months. Reliability was assessed using Cronbach’s alpha. Construct and discriminant validity was assessed by exploratory factor analysis and known-group comparison.

**RESULTS:**

The exploratory factor analysis revealed a three-dimensional structure, explaining 63.3% of the total variance. The factors were labelled ‘Own capacity’, ‘Professional support’ and ‘Decision making’. No items were excluded. Internal consistency was demonstrated with an overall Cronbach’s alpha of 0.94 for the total scale. Primiparous women, women who had an emergency cesarean section, and women who had been exposed to the Kristeller manoeuvre had a lower overall score on the CEQ-SK compared to parous women, women having a vaginal birth and women not exposed to the Kristeller manoeuvre.

**CONCLUSION:**

The CEQ-SK was found to be a valid and reliable tool for evaluating childbirth experience in Slovakia. The original CEQ is a four-dimensional questionnaire; however, factor analysis showed a three-dimensional structure in the Slovak sample. This needs to be taken into consideration when comparing the results from the CEQ-SK with studies that use the four-dimensional structure.

## INTRODUCTION

Childbirth is one of the most significant experiences in a woman’s life^1^. A positive childbirth experience can enable women to grow and feel empowered^2,3^. However, some women describe childbirth as a negative experience^3^, and instead of feeling empowered, they report a feeling of helplessness and a lack of control^3^. A negative childbirth experience may have short- and long-term impacts on women’s well-being, health, transition to motherhood and future reproduction^4-6^.

Factors that may influence the birth experience include expectations about the experience, information, complications, care, communication, feeling of control, and perception of pain^2-4,7-9^. Women’s subjective interpretations of the birth experience are not necessarily related to actual adverse events^10^. When women feel safe and well taken care of during birth, the overall experience can be positive, despite complications^9,11^. Non-clinical aspects of labor and childbirth care, such as the provision of emotional support through labor companionship, effective communication and respectful care, are essential components of the experience of care^12^ and important during pregnancy and birth^13^. Unfortunately, thousands of women experience disrespectful care, such as being neglected, not receiving comfort or pain relief, and even being subject to verbal and physical abuse^14^. Disrespect, abuse and violence against women during pregnancy and childbirth is a violation of fundamental human rights^15-17^. Therefore, using validated and reliable instruments to examine women’s birth experiences is important to ensure and promote respectful and empathetic care^18^.

Different instruments have been used to examine women’s childbirth experiences, and a recent systematic review^18^ identified the childbirth experience questionnaire (CEQ)^19^ as an instrument that provides valid scores and systematically evaluates women’s experiences^18^. The CEQ has been translated and evaluated in several countries^20-24^, and a revised version (CEQ2) has been validated in Sweden and the UK^25,26^.

To our knowledge, few studies have examined women’s experiences with care during childbirth in Slovakia^27-32^. Maskálová et al.^28^ investigated women’s satisfaction with childbirth by using a translated, but not validated version of the CEQ. They included 161 primiparous women and found that the overall level of satisfaction with childbirth was relatively low. Women were more satisfied after an operative delivery compared with a spontaneous birth, and they were least satisfied with the perceived professional support. Qualitative investigations on women’s birthing experience in Slovak healthcare facilities, published by Citizen, Democracy and Accountability (CDA) in 2015, implied that serious violations of women’s human rights occurred in connection with childbirth in Slovakia^29^. The violations included: a lack of informed consent with interventions during childbirth; interventions carried out despite refusal; lack of information provided to women before and during childbirth; interventions carried out routinely, e.g. episiotomies, Kristeller manoeuvre (fundal pressure during the second stage of labor) without sufficient or any pain relief; denial of a companion of choice present during all stages of childbirth; inability of women to move freely and choose the birthing position; grave violations with regard to privacy, intimacy and personal integrity; and undignified, disrespectful and abusive behavior by healthcare staff^29^. A follow-up qualitative investigation from 2016 supported these findings^30^, as did a report from 2020^32^ and a study by the Slovak Public Defender of Rights (the ombudsperson) in 2021^31^. A cross-sectional study in 2020 investigating the satisfaction of Slovak women with psychosocial aspects of perinatal care concluded that there was a need for various interventions in the childbirth care system, including the provision of emotional support and empowered decision making for birthing women^27^.

### Childbirth care in Slovakia

In Slovakia, no childbirth care other than that provided in hospitals is guaranteed, and the system is medicalised^30,32^. Obstetric authorities determine the nature of care provided both in individual healthcare facilities and at the state level^30,32^. Although midwives are employed in all maternity wards and Slovak legislation allows for independent childbirth assistance by midwives, in most of the cases, they do not provide childbirth care independently but under the leadership of obstetricians^30^. Until 2021, when the Ministry of Health published guidelines on peri- and postpartum hemorrhage, on care in low-risk pregnancy and on care in low-risk birth^33^, there had not been any guidelines on birth care adopted at the state level^30-32^.

Due to a lack of studies and validated instruments to evaluate women’s childbirth experiences in Slovakia, we wished to adapt a well-known tool used to understand women’s childbirth experiences and evaluate the quality of care^28^. Therefore, the aim of this study is to adapt and validate the CEQ in Slovakia.

## METHODS

### Childbirth Experience Questionnaire (CEQ)

The CEQ was developed in Sweden by Denker et al.^19^ in 2010 and validated with 920 primiparous women^19^, and an English version of the CEQ was validated in the UK in 2015^20^. The CEQ includes 22 items and was originally used to assess women’s first childbirth experiences^19^ in four main domains: Own capacity, Professional support, Perceived safety, and Participation. A revised version, the CEQ2, which contains more relevant items regarding information and decision making, was validated in Sweden and the UK in 2020^25,26^.

To be an appropriate tool for evaluating maternity care in Slovakia, the CEQ-SK was developed in 2019 from a combination of the CEQ and the CEQ2 to eventually include 22 items (Supplementary file). Some items were modified to adapt to the childbirth context provided in Slovakia. For example, a CEQ2 item on the treatment of both a woman in childbirth and her partner was modified to cover any companion of a woman’s choice and split into two questions to avoid confusion in situations in which a companion was not present or was treated differently from the birthing woman. Similarly, questions that originally referred to treatment by midwives were modified to cover treatment by healthcare staff in general, since midwives are not the primary providers in Slovak birthing facilities and healthcare staff often do not introduce themselves^29,30^. Hence, women do not know whether a particular staff member providing care is a midwife, a nurse or a doctor.

Responses to 19 items were scored using a 4-point Likert scale ranging from 1 (totally disagree) to 4 (totally agree). Three items referring to perceived pain, sense of control and sense of security were assessed using a visual analogue scale (VAS), where zero indicated no pain/no control/no security and 100 indicated the worst imaginable pain/complete control/feeling of complete security. The VAS scores were transformed to categorical values: 0–40=1, 41–60=2, 61–80 =3, and 81–100=4. The scores of negatively worded items were reversed. Higher CEQ-SK scores mean a more positive childbirth experience.

The CEQ-SK was part of a larger questionnaire, with additional questions referring to some specific aspects of human rights in the context of childbirth care, as provided in Slovakia. The survey was carried out by CDA, a human rights non-governmental organization based in Slovakia. In the process of designing the CEQ-SK and in data collection, CDA cooperated with Women’s Circles (an NGO, Slovakia).

### Translation procedure

After obtaining permission to use and adapt the CEQ/CEQ2 from Anna Dencker (personal communication, 26 June 2019 and 14 May 2020), we modified the questionnaire as described above and in the Supplementary file, and translated the new CEQ-SK into the Slovak language. First, the CEQ-SK was translated from English into Slovak by one of the Slovak team members and then two other members of the Slovak team proofread and edited this translation. The questionnaire was back translated into English by a native speaker.

Comprehension of the Slovak version of the CEQ was tested by women during the first pre-test (28 women) and later during the second pre-test (22 women), and subsequently revised (see below for more detail).

### Validation study

*Settings, participants and procedure*

In addition to the CEQ-SK questions, women answered questions about demographics that included age, education level, and the region in Slovakia where they gave birth. They answered questions related to their last delivery in a Slovakian hospital, which included information about parity, gestational age at birth, mode of delivery, use of epidural, episiotomy, and use of the Kristeller manoeuvre.

The full version of the questionnaire (containing the CEQ-SK questions, the demographic questions and the other questions not reported in this article) was administered online through the Netquest-e-platform and posted on different sites: the CDA website, the Women’s Circles website and their Facebook site, the Aspekt website and websites of other NGOs, and on women’s e-media operating in Slovakia (SME Žena, Ahojmama, and Pravda). The data were collected from October 2019 to November 2019. A total of 918 women answered the questionnaire.

In this study, 513 women who gave birth within a period of one to six months prior to answering the questionnaire were included. We excluded 198 women who had a planned cesarean section and 29 with incomplete data, leaving a sample of 286 women.

*Face validity*

At the first stage of the pre-test, a group of nine women from Bratislava who had given birth in a healthcare facility in Slovakia in a period no longer than two years before the pilot testing were asked to come to an agreed place and fill in the CEQ-SK online. Afterwards, a member of the team asked questions about the content and the way the questions in the questionnaire were phrased and whether they were understandable and acceptable to the members of the pre-test group. Another 19 women, recruited on the basis of the same criteria, filled in the CEQ-SK online with these extra questions about comprehension and fluency being asked at the end of the questionnaire, leading to the following changes: The answer option: ‘It does not concern me’ was added to items Q9, Q10, Q12 and Q14. In addition, we included a general emphasis in the questionnaire that ‘childbirth’ covered all of its stages (the Slovak language does not have specific terms for ‘labor’ and ‘delivery’, and during the face-to-face testing, some women only considered the pushing stage as childbirth).

Second, after incorporating the comments on the questionnaire received from the women from the first stage of the pre-test, the new version of CEQ-SK was tested (the second stage of the pre-test). This version was tested by a group of 22 women (the same criteria as above) who completed the CEQ-SK online, again with extra questions about comprehension and relevance at the end of the questionnaire.

*Item characteristics*

Descriptive statistics were computed to characterize item score distribution, including the mean scores of the CEQ-SK questions. Means (range: 1–4) for a given item were calculated without the answer ‘It does not concern me’ that was added as an answer option in items Q9, Q10, Q12 and Q14.

*Construct validity*

The construct validity of the CEQ-SK was assessed by exploratory factor analysis using principal component analysis as a method of extraction. Oblique rotation (promax) was conducted. The Kaiser rule (eigenvalue >1.0) was applied to determine the number of factors to extract.

*Discriminant validity*

The discriminant validity of the CEQ-SK was assessed using the Mann-Whitney U-test by comparing scores from subgroups known to differ for childbirth experience^30^. The compared variables of CEQ-SK included parity, use of epidural, mode of delivery, episiotomy, and Kristeller manoeuvre.

### Ethics considerations

This study was planned as an anonymous study via a platform such that not to register or store any data that can identify the respondents; hence it did not require ethical approval. The study was assessed by The Board of Trustees of CDA, an independent supervising body also serving as the ethics committee for the purposes of research and monitoring carried out by CDA, and they confirmed this. A short informative text that provided information about the purpose of the study was contained in the introduction to the questionnaire. The participants also gave their explicit consent to use all the data contained in their answers. The participants’ IP addresses were not registered, and to ensure that they were not identifiable, the background information was general and limited.

## RESULTS

The total sample in this validation study was 286 women and their characteristics can be seen in Table 1. Most women were aged between 26 and 35 years, and all regions of Slovakia were represented. Approximately 60% of the women were primiparous. Most of the respondents had a spontaneous onset of labor (71%).

**Table 1:** Demographic and clinical data of women in the validation study of CEQ-SK (N=286)

*Variable*	*n (%)*
**Maternal age** (years)	
18–25	34 (11.9)
26–35	219 (76.6)
36–41	33 (11.5)
**Regional representation**	
Bratislava	113 (39.5)
Trnava, Nitra,Trenčín	52 (18.2)
Žilina, BanskáBystrica	62 (21.7)
Košice, Prešov	59 (20.6)
**Gestational age in weeks**	
≤37	27 (9.4)
38–41	219 (76.6)
≥42	38 (13.3)
**Parity**	
Primiparas	168 (58.7)
Multiparas	118 (41.3)
**Onset of labor**	
Spontaneous	203 (71.0)
Induced	76 (26.6)
**Delivery**	
Vaginal	247 (86.4)
Emergency cesarean	39 (13.6)
Epidural	85 (29.7)
Episiotomy	109 (38.1)
Kristeller manoeuvre	79 (27.6)

Table 2 gives an overview of the item characteristics with means and standard deviations of the CEQ-SK item responses. Items concerning how women’s companions were treated obtained the highest scores. Being able to choose the birthing position during delivery returned the lowest score (Table 2).

**Table 2:** Information of 22 items and mean score in CEQ-SK

*Items*	*Total sample per item*	*Mean (SD)*
Q1R -Labor and birth went as I had expected.	286	2.90 (0.94)
Q2 - I felt scared during labor and birth.	286	2.64 (1.01)
Q3 - I felt capable during labor and birth.	286	3.33 (0.84)
Q4R - I was tired during labor and birth.	286	2.29 (1.14)
Q5 - I felt happy during labor and birth.	286	2.54 (0.93)
Q6 - I felt that I handled the situation well.	286	2.93 (0.91)
Q7R - I wish the staff had listened to me more during labor and birth.	286	2.76 (1.06)
Q8 - I took part as much as I wanted in decisions regarding my care and treatment.	286	3.02 (0.98)
Q9 - During labor, I could change my position at any time, deciding whether I would stand, lie, kneel or squat.	261	2.59 (1.09)
Q10 - I could decide for myself on the form of relief from labor pains in the hospital (e.g. massage, hot water, change of position, epidural).	259	3.03 (1.03)
Q11 - I was treated with kindness and respect.	286	3.35 (0.86)
Q12 – I could decide for myself in which position I finally gave birth to my child (whether I would stand, lie, kneel or squat).	243	1.98 (1.11)
Q13 - I received all the information I needed during labor and birth.	286	3.12 (0.91)
Q14 - The person(s) accompanying me was (were) treated with kindness and respect.	246	3.56 (0.75)
Q15 - I have many positive memories from childbirth.	286	2.94 (0.99)
Q16R - I wish the medical staff had given me more care and understood my needs better.	286	2.72 (1.00)
Q17 - My impression of the team’s medical skills made me feel secure.	286	3.35 (0.83)
Q18R - I have many negative memories from childbirth.	286	3.22 (0.95)
Q19R - Some of my memories from childbirth make me feel depressed.	286	3.27 (1.03)
Q20R^a^ - On the whole, how painful did you feel childbirth was?	286	2.30 (1.05)
Q21^a^ - On the whole, how much control did you feel you had over decision making during childbirth?	286	2.07 (1.05)
Q22^a^ - On the whole, how secure did you feel during childbirth?	286	3.02 (1.05)

R: ratings of negatively worded statements are reversed.

a Visual analogue scale (VAS).

### Face validity

Both pre-tests showed good face validity, and the CEQ-SK was acceptable and understandable to the women.

### Construct validity

The 22 items of the CEQ-SK were subjected to exploratory factor analysis (Table 3). All items with factor loadings higher than 0.3 are shown. No items were excluded from the CEQ-SK. The analyses revealed three factors that explained 63.3% of the total variance. The factors were labelled:‘ Own capacity’ (46.6%), ‘Professional support’ (10.1%), and ‘Decision making’ (6.6%).

**Table 3:** Factor loadings, eigenvalues and explained variance after exploratory factor analysis in the validation study of CEQ-SK (N=286)

*Item*	*Factors*		
	*1*	*2*	*3*
**Factor 1: Own capacity**			
Q4R - I was tired during labor and birth.	**0.880**	-0.330	
Q5 - I felt happy during labor and birth.	**0.794**		
Q3 - I felt capable during labor and birth.	**0.789**		
Q6 - I felt that I handled the situation well.	**0.781**		
Q20RA – On the whole, how painful did you feel childbirth was?	**0.768**	-0.383	
Q2R - I felt scared during labor and birth.	**0.721**		
Q18R - I have many negative memories from childbirth.	**0.713**	0.319	
Q19R - Some of my memories from childbirth make me feel depressed.	**0.672**		
Q15 - I have many positive memories from childbirth.	**0.664**		
Q1 - Labor and birth went as I had expected.	**0.580**		
**Factor 2: Professional support**			
Q16R - I wish the medical staff had given me more care and understood my needs better.		**0.944**	
Q14 - The person(s) accompanying me was (were) treated with kindness and respect.		**0.920**	
Q11 - I was treated with kindness and respect.		**0.825**	
Q7R - I wish the staff had listened to me more during labor and birth.		**0.821**	
Q13 - I received all information I needed during labor and birth.		**0.706**	
Q17 - My impression of the team’s medical skills made me feel secure.		**0.670**	
Q22RA - As a whole, how secure did you feel during childbirth?	0.405	**0.491**	
Q8 - I took part as much as I wanted in decisions regarding my care and treatment.		**0.448**	0.362
**Factor 3: Decision making**			
Q12 - I could decide for myself in which position I finally gave birth to my child ( whether I would stand, lie, kneel or squat ).			**0.901**
Q9 - During labor, I could change my position at any time, deciding whether I would stand, lie, kneel or squat.			**0.823**
Q10 - I could decide for myself on the form of relief from labor pains in the hospital ( e.g. massage, hot water, change of position, epidural ).			**0.822**
Q21RA – On the whole, how much control did you feel you had over decision making during childbirth?			**0.584**
**Eigenvalue**	10.2	2.2	1.4
**Variance explained (%)**	46.6	10.1	6.6
**Cumulative variance explained (%)**	46.6	56.7	63.3

Extraction method: principal component analysis. The items are collected within the given factors based on the bold values. Factor loadings <0.30 are not shown.

Internal consistency (measured with Cronbach’s alpha) of the CEQ-SK was 0.90, 0.91 and 0.82 for ‘Own capacity’, ‘Professional support’, and ‘Decision making’, respectively. Cronbach’s alpha for the total scale was 0.94 (Table 4).

**Table 4:** Descriptive statistics for subscales and total scale scores in the validation study of CEQ-SK (N=286)

*Subscale*	*n*	*Number of items*	*Range*	*Mean (SD)*	*Cronbach’s alpha*
Own capacity	286	10	1.00–4.00	2.84 (0.71)	0.90
Professional support	246	8	1.13–4.00	3.14 (0.72)	0.91
Decision making	230	4	1.00–4.00	2.43 (0.87)	0.82
Total scale	206	22	1.14–3.95	2.87 (0.65)	0.94

### Discriminant validity

Known-group validation was used to assess discriminant validity (Table 5). Women who had a vaginal birth had significantly higher scores on the subscales: ‘Own capacity’ and ‘Professional support’ and an overall higher CEQ-SK score than women who had an emergency cesarean section. Women with spontaneous onset of labor scored higher than women who had labor induced in all subscales, but the differences were not significant. Multiparous women had higher scores than nulliparous women for ‘Own capacity’, as well as for the overall CEQ-SK score. As shown in Table 5, women who had an epidural scored lower on the ‘Own capacity’ subscale than women who did not use it. Women who had an episiotomy or who were subject to the Kristeller manoeuvre had significantly lower scores in all subscales as well as overall, except for ‘Professional support’ in the case of episiotomy, where the difference was not significant.

**Table 5:** Mean differences in subscales and total scores between groups in the validation study of CEQ-SK (N=286)

*Parity*	*Own capacity*		*Professional support*		*Decision making*		*Total score*	
	*n*	*Mean (SD)*	*n*	*Mean (SD)*	*n*	*Mean (SD)*	*n*	*Mean (SD)*
Primiparas	168	2.72 (0.71)	145	3.09 (0.75)	136	2.36 (0.85)	125	2.77 (0.66)
Multiparas	118	3.01 (0.68)	101	3.22 (0.68)	94	2.53 (0.89)	81	3.02 (0.62)
p		**0.000**		0.183		0.145		**0.006**
**Onset of labor**								
Spontaneous	203	2.89 (0.70)	177	3.16 (0.74)	163	2.49 (0.89)	147	2.92 (0.66)
Induced	76	2.75 (0.74)	67	3.10 (0.69)	64	2.31 (0.80)	57	2.75 (0.65)
p		0.179		0.334		0.146		0.146
**Type of delivery**								
Vaginal	247	2.90 (0.69)	217	3.19 (0.69)	215	2.44 (0.88)	191	2.91 (0.63)
Cesarean	39	2.44 (0.67)	29	2.75 (0.85)	15	2.28 (0.78)	15	2.38 (0.73)
p		**0.000**		**0.009**		0.460		**0.007**
**Use of epidural**								
Yes	85	2.66 (0.77)	80	3.18 (0.71)	72	2.39 (0.85)	69	2.76 (0.68)
No	197	2.93 (0.66)	165	3.13 (0.72)	157	2.45 (0.88)	137	2.93 (0.64)
p		**0.010**		0.560		0.577		0.106
**Episiotomy**								
Yes	109	2.72 (0.75)	94	3.09 (0.69)	94	2.22 (0.86)	83	2.73 (0.66)
No	171	2.91 (0.68)	147	3.18 (0.75)	130	2.60 (0.85)	118	2.97 (0.64)
p		**0.036**		0.125		**0.001**		**0.007**
**Kristeller manoeuvre**								
Yes	79	2.54 (0.69)	73	3.01 (0.72)	73	2.23 (0.78)	68	2.62 (0.63)
No	192	2.97 (0.69)	162	3.21 (0.71)	150	2.54 (0.90)	132	3.01 (0.64)
p		**0.000**		**0.019**		**0.011**		**0.000**

## DISCUSSION

In this study, we adapted and validated the CEQ in the Slovak context. The CEQ-SK was found to be a valid and reliable tool for evaluating childbirth experiences in Slovakia. The original CEQ is a four-dimensional questionnaire; however, factor analysis revealed a three-dimensional structure in the Slovak sample. The factors were labelled: ‘Own capacity’, ‘Professional support’ and ‘Decision making’. A high internal consistency was demonstrated, with a Cronbach alpha between 0.82 and 0.94. The Cronbach alphavalues found in this study are consistent with other studies that have validated the CEQ^19,20,22,24^.

Similar to the study by Boie et al.^24^, which validated the use of the CEQ in Denmark, we found a three-dimensional model slightly different from the original four-dimensional model shown in both studies by Dencker et al.^19,25^ and by other studies that have validated the CEQ^21-23^. Most items originally within the domain ‘Perceived safety’ (items 2, 18, 19, 15) were grouped with the ‘Own capacity’ domain in our model, and items related to feeling secure (items 17 and 22) were grouped with the domain ‘Professional support’. Hence, the domain ‘Perceived safety’, which is seen in both versions of the original CEQ, did not appear in the factor analysis in the Slovak sample. A similar pattern was seen in the study from Denmark^24^, where the domains ‘Own capacity’ and ‘Perceived Safety’ merged into one domain named ‘Own capacity’. Interestingly, one of the items regarding safety in the Danish study also fell into the ‘Professional support’ domain. However, it needs to be considered that the study from Denmark used a questionnaire that was similar to the original CEQ, whereas we used an adapted version that combined the original and second versions. It should also be considered that Denmark and Slovakia have different childbirth care systems.

There may be several reasons for this particular outcome of the CEQ-SK. The questionnaires were used in different cultural and language contexts than the previous CEQs, and it may be the case that issues of women’s feelings and memories that were asked in items 2, 18, 19, and 15 were perceived as any other issues of feelings and memories, including those asked in items 4, 5, 3, 6, and 20, thus leading to their grouping together in one domain. However, the loading of these into the ‘Own capacity’ domain may also reflect the high degree of normalization of harmful practices and other violations of human rights present in the Slovak childbirth care system^29,30^. Obstetricians have a strong position within the system, and a high degree of authoritative knowledge is attributed to them in Slovakia^30^. This contributes not only to hospital practices, procedures and behaviors being normalized by childbirth care providers but also among some birthing women and the general population^29,30,34,35^. This may partially explain why issues that could normally fall within a relatively free-standing concept of ‘safety’, as was the case of the original CEQ with items 2, 18, 19 and 15, emerged as a matter of women’s ‘Own capacity’. On the other hand, it needs to be emphasized that without regard to whether and to what extent women may tend to normalize and internalize the values and practices promoted by the current childbirth care system in Slovakia, the loading of items 17, 18 and 22 into the ‘Professional support’ domain may indicate that women do understand the roles and responsibilities of the healthcare staff with regard to women’s feelings of security during childbirth, as well as with regard to the potential negative memories resulting from their childbirth.

We decided to rename ‘Participation’ to ‘Decision making’ for this domain. The main reason for this is the concept of informed consent/informed decision making, which is a prerequisite for any intervention in childbirth care^36,37^. The concept of autonomous decision making, grounded in the right to privacy and to personal autonomy, also applies to all other aspects and circumstances of childbirth care, such as the choice of a birth companion or the choice of the birthing position. Hence, a reference to ‘Participation’ may not sufficiently reflect the fact that it is the laboring women who should be the main actors and ultimate decisionmakers regarding their labor and birth.

The known-group validation is in line with previous studies that used different versions of the CEQ. The multiparous women in this study had a significantly higher total score on the CEQ-SK than primiparous women, in line with the study by Dencker et al.^25^ from 2020 and other studies that included both primiparous and multiparous women^21,22^. Women who had an emergency cesarean section had a lower overall score than women with a vaginal birth, and they scored significantly lower in two of the three dimensions (‘Own capacity’ and ‘Professional support’). Women who had operative births were known to score lower using the CEQ^21,24^. In the original study by Denkcer et al.^19^, and in the studies by Kalok et al.^23^ and Boie et al.^24^, women with operative birth scored lower in all domains^19,23,24^. We cannot compare their results directly with ours, as they included both emergency cesarean sections and operative vaginal births, and our study looks at emergency cesarean sections only. Having a vaginal operative birth is not that common in Slovakia, and cesarean sections are performed in most cases when an emergency occurs in birth^38^.

Women who had been exposed to the Kristeller manoeuvre scored significantly lower in all domains and had a low overall score in the CEQ-SK. The Kristeller manoeuvre was commonly used in our sample (27.6%), and other studies that have been undertaken in Slovakia confirm its common occurrence^29-32^. According to a survey carried out by the Slovak ombudsperson, the intervention occurred in 39.6% of the surveyed cases in 2017, 37.8% in 2018, and 34.7% in 2019^31^. The official data for this procedure, based on information collected solely from healthcare facilities, was only 0.43% of all childbirths for 2017^38^, and for subsequent years, this data stopped being collected for official statistical purposes^32,33^, with the explanation that the procedure was prohibited as it was not grounded in evidence-based medicine^31^. This may illustrate a serious problem with the continued use of procedures in Slovak childbirth facilities that are not evidence-based and that can negatively impact women’s childbirth experiences. The strikingly high discrepancy between official statistical data, based on data received from healthcare facilities^38^, and data from other sources^29-32^, albeit non-representative yet collected from women who have received childbirth care, confirm the need for the healthcare system to place more emphasis on basing the collection of childbirth-related data also on women’s experience.

### Strengths and limitations

We studied a sample size of 286 women, guided by a subject-to-item ratio of 1:10, which is a prevalent recommendation for determining a sample size when a health instrument is being validated^39^. Recruitment via social media could represent a limitation of this study. A survey on social media may attract women who are not representative of the general population. Even though most of the population makes use of social media, we may have failed to reach some parts of the population. However, studies have shown that recruitment through social media can provide representative samples that match traditional data-collection methods^40^. The emergency cesarean section prevalence in our study of 13.6% is similar to the cesarean section rate in Slovakia from 2019 (12.3%)^38^. In this study, more primiparous women participated than parous women (58.7% vs 41.3%). Of all the women who gave birth in Slovakia in 2019, 24874 (44%) were primipara^38^. This may have affected the CEQ-SK scores.

Any retrospective cross-sectional study runs the risk of potential recall bias and women answered the CEQ-SK between one and six months after birth. The time difference could have influenced how the women recalled their births. However, women tend to rate their experiences more positively during the first week after birth, and after 6 weeks 50% change perception and have a lower CEQ score. This is the memory that usually follows them, and they are considered stable over time^41^. The clinical data were self-reported and not based on the patients’ records, which could have affected the accuracy of the data. In this study, we found that using a three-dimensional model, different from the original four-dimensional model, limited the comparability of CEQ scores with studies using the four-factor model. In addition, the CEQ-SK was modified using both CEQ and CEQ2.

## CONCLUSIONS

The CEQ-SK was found to be a valid and reliable tool for evaluating childbirth experience in Slovakia. The original CEQ is a four-dimensional questionnaire; however, factor analysis showed a three-dimensional structure in the Slovak sample. This needs to be taken into consideration when comparing the results from the CEQ-SK with studies that use the four-dimensional structure.

## Data Availability

The data supporting this research are available from the authors on reasonable request.
