# Supplementary material for: Adaption and validation of the childbirth experience questionnaire (CEQ-SK) in Slovakia
Source: Eur J Midwifery. 2023 Mar 14;7:6. doi: 10.18332/ejm/160973 (PMC10012084; doi:10.18332/ejm/160973)
Supplement: Supplementary file 1 [file EJM-7-6-s1.pdf]

Box 1. Slovak version of the childbirth experience questionnaire (CEQ-SK)

**CEQ-SK**	**CEQ version**
Q1 - Labour and birth went as I had expected.	1+2
Q2 - I felt scared during labour and birth.	1+2
Q3 - I felt capable during labour and birth.	1+2
Q4 - I was tired during labour and birth.	1+2
Q5 - I felt happy during labour and birth.	1+2
Q6 - I felt that I handled the situation well.	1+2
Q7 - I wish the staff had listened to me more during labour and birth.	2
Q8 - I took part as much as I wanted in decisions regarding my care and treatment.	2
Q9 - During labour, I could change my position at any time, deciding whether I would stand, lie, kneel or squat.	1, M
Q10 - I could decide for myself on the form of relief from labour pains in the hospital (e.g. massage, hot water, change of position, epidural).	1
Q11 - I was treated with kindness and respect.	2, M
Q12 – I could decide for myself in which position I finally gave birth to the child (whether I would stand, lie, kneel or squat).	1, M
Q13 - I received all the information I needed during labour and birth.	2
Q14 - The person(s) accompanying me was (were) treated with kindness and respect.	2
Q15 - I have many positive memories from childbirth.	1+2
Q16 - I wish the medical staff had given me more care and understood my needs better.	2, M
Q17 - My impression of the team's medical skills made me feel secure.	1+2
Q18 - I have many negative memories from childbirth.	1+2
Q19 - Some of my memories from childbirth make me feel depressed.	1+2
Q20 [a] - As a whole, how painful did you feel childbirth was?	1+2
Q21 [a] - As a whole, how much control did you feel you had over decision making during childbirth?	1+2, M
Q22 [a] - As a whole, how secure did you feel during childbirth?	1+2

1 = CEQ version 1, 2 = CEQ version 2 and M = modified

[a] Visual analogue scale (VAS)

No items were excluded after the analysis.

© 2023 Henriksen L. et al.
